# Prognostic Factors of Melanoma Patients with Satellite or In-Transit Metastasis at the Time of Stage III Diagnosis

**DOI:** 10.1371/journal.pone.0063137

**Published:** 2013-04-29

**Authors:** Benjamin Weide, Christine Faller, Petra Büttner, Annette Pflugfelder, Ulrike Leiter, Thomas Kurt Eigentler, Jürgen Bauer, Andrea Forschner, Friedegund Meier, Claus Garbe

**Affiliations:** 1 Center for Dermatooncology, Department of Dermatology, University Medical Center, Tübingen, Germany; 2 Skin Cancer Research Group, School of Public Health, Tropical Medicine and Rehabilitation Sciences, James Cook University, Townsville, Australia; Istituto Superiore di Sanità, Italy

## Abstract

**Background:**

Prognosis of patients with loco-regional skin metastases has not been analyzed in detail and the presence or absence of concurrent lymph node metastasis represents the only established prognostic factor thus far. Most studies were limited to patients already presenting with skin lesions at the time of initial diagnosis. We aimed to analyze the impact of a broad penal of prognostic factors in patients with skin metastases at the time of first metastatic spread, including patients with synchronous lesions already present at the time of initial diagnosis, stage I/II patients with loco-regional recurrence and patients initially presenting with skin metastasis but unknown primary melanoma.

**Patients and Methods:**

We investigated disease-specific survival of 380 patients treated at our department between 1996 and 2010 using Kaplan Meier survival probabilities and Cox-proportional hazard analysis.

**Results:**

Five-year survival probability was 60.1% for patients with skin metastases only and 36.3% for those with synchronous nodal metastases. The number of involved nodes and a tumor thickness of at least 3 mm had independent negative impact on prognosis. A strong relationship was identified between the risk of death and the number of involved nodes. Neither ulceration nor the timing of the first occurrence of metastases as either in stage I/II patients, at the time of excision of the primary melanoma or initially in patients with unknown primary tumor, had additional effects on survival.

**Conclusion:**

Lymph node involvement was confirmed as the most important prognostic factor for melanoma patients with loco-regional skin metastasis including those with unknown primary tumor and stage I/II patients with skin recurrence. Consideration of the tumor thickness and of the number of involved lymph nodes instead of the exclusive differentiation into presence vs. absence of nodal disease may allow a more accurate prediction of prognosis for patients with satellite or in-transit metastases.

## Introduction

The prognosis of melanoma patients with loco-regional metastasis varies widely with 5-year survival rates ranging between 39% and 70% [1], [2]. The most comprehensive investigation of prognostic factors of melanoma patients with loco-regional metastasis was conducted by the American Joint Committee on Cancer (AJCC) who released the 7^th^ edition of the classification recommendation in 2009 [1]. The recommendation was based on the multivariable analysis of more than 2900 patients with lymph node metastasis and included the number of tumor-bearing nodes, the tumor burden (microscopic vs. macroscopic) and ulceration of the primary melanoma to assign patients to the different prognostic sub-stages IIIA-C.

In contrast, prognosis of patients with loco-regional skin metastases has not been analyzed in detail and the concurrent presence or absence of lymph node metastasis represents the only established prognostic factor thus far.

Patients with skin metastases but without lymph node involvement were aligned to a distinct N category N2c (sub-stage IIIB) based on a survival analysis of 399 melanoma patients [1]. Patients with combined lymph node and skin metastases showed a worse prognosis and were defined as N3 and therefore classified as sub-stage IIIC, based on a similar prognosis compared to patients with 4 or more involved lymph nodes but no skin lesions. A detailed analysis of prognostic markers in this patient cohort was not published thus far. Other previous analyses were restricted to patients receiving limb perfusion [3], [4] to those with recurrences [5], [6] or were only performed in small cohorts of patients [7]. The limited knowledge about prognosis of patients with skin metastases hampers not only patient counseling but also led to the exclusion of these patients in many clinical trials particularly in the adjuvant setting [8]–[10].

Aim of this study was to identify prognostic factors in melanoma patients with satellite or in-transit metastasis at the time of stage III diagnosis in addition to the presence or absence of concurrent lymph node involvement to allow a more accurate prediction of prognosis.

## Methods

### Ethics statement

All had given their written informed consent to have clinical data recorded by the Central Malignant Melanoma Registry (CMMR) registry. The institutional ethics committee Tübingen approved the study (ethic vote 711/2012R).

### Patients

Patients with skin metastases treated between 1996 and 2010 at the University Department of Dermatology in Tübingen, Germany, were identified in the Central Malignant Melanoma Registry (CMMR) database which prospectively records patients from more than 60 dermatological centers in Germany. The aims and methods of data collection by the CMMR have previously been reported in detail [11]. Only patients with loco regional skin metastases or a combination of loco regional skin and lymph node metastasis at the time point of first metastatic spread were included into this analysis. Patients with prior loco-regional lymph-node metastasis, or patients with concurrent or prior distant metastasis were excluded. In this study, a skin metastasis was defined to be localized either dermal or subcutaneously in the anatomical region between the primary melanoma and the next proximal lymph node basin (groin, axilla or cervical region). Patients with satellite- and/or in-transit metastases were eligible, while those with isolated true local recurrences resulting from an incompletely excised primary tumor were excluded. Patients with unknown primary melanoma were eligible if skin lesions were confined to one anatomical region (e.g. one extremity) but were excluded, if there was evidence of metastatic disease in more than one region with a main lymph node basin in between (e.g. trunk and one extremity). 380 patients were finally included after individual file review.

Data obtained for each patient included gender, age at stage III diagnosis (<60 vs. 60–69 vs. ≥70 years), situation at stage III diagnosis (immediately metastasized melanoma vs. loco-regionally relapsed melanoma vs. melanoma with unknown primary tumor [MUP]), presence and number of synchronous lymph node metastases (0 vs. 1 vs. 2–3 vs. 4 or more), date of the last follow-up, and the date and cause of death, if applicable. The following characteristics of the primary tumor were analyzed: body site (axial vs. extremities), Breslow's tumor thickness (<3 mm vs. ≥3 mm), Clark's level of invasion (I–III vs. IV vs. V), ulceration (yes vs. no), and subtype (superficial spreading melanoma vs. nodular melanoma vs. lentigo maligna melanoma vs. acral lentiginous melanoma).

### Statistical analysis

Follow-up time was defined from the date of diagnosis of the first skin metastasis to the date of last follow-up or death. Estimates of cumulative survival probabilities according to Kaplan-Meier were described together with 95% confidence intervals (CIs) and compared using two-sided log-rank test statistics. Median survival times (MST) are presented. For the analysis of disease-specific survival, patients who were alive at the last follow-up or died without evidence of metastatic melanoma were considered to be censored cases, while patients who had died due to melanoma were considered an “event”.

Multivariable Cox proportional hazard models were used to determine independent prognostic factors. Categorized variables were dummy coded to adhere to the linearity assumption of multivariable regression analysis. All characteristics described above were considered in the Cox regression models. Missing values were assessed independently as a separate group to allow the inclusion of patients with MUP. Forward and backward stepwise procedures of the multivariable modeling process were conducted. Results of the Cox models were described by means of hazard ratios together with 95% CIs, p-values were based on the Wald test.

Confounding was assessed by checking the effect of each remaining non-significant variable, which was not in a model, on factors in the model. If changes to the regression coefficient of a factor in the model of 5% or more occurred, then the respective variable was considered a confounder and the model was adjusted for it. Throughout the analysis, p values less than 0.05 were considered as statistically significant. All statistical analyses were carried out using the SPSS Version 19 (IBM SPSS, Chicago, Illinois, USA).

## Results

### Description of sample

Patient characteristics are shown in Table 1. A total of 380 melanoma patients (49% male) were included in the survival analysis at the time of initial stage III diagnosis. The median age was 66 years (inter quartile range [IQR] 55–73 years). The median follow-up time for patients who died was 25 months (IQR: 14–44) and 56 months (IQR: 26–102) for patients who were alive at the last date of observation. The median survival time according to Kaplan Meier (MST) was 74 months. Cumulative survival rates were 92.5% (1 year), 77.7% (2-years), 54.6% (5-years) and 42.1% (10-years). Overall, 50.5% of patients developed the first loco-regional metastasis after the initial excision of the primary melanoma in contrast to 34.5% who already presented with metastases at the initial diagnosis and 15.0% who had an unknown primary tumor.

**Table 1:** Patient characteristics and survival analysis according to Kaplan-Meier.

Factor	n	%	% Dead	5 Years survival rate [95%-CI*] (%)		p **
Gender						
Male	185	48.7	44.3	51.0	[42.8; 59.2]	0.225
Female	195	51.3	41	57.9	[50.1; 65.7]	
Age						
≤60 years	132	34.7	48.5	52.5	[43.1; 61.9]	0.834
61–70years	119	31.3	42.9	58.0	[48.0; 68.0]	
>70 years	129	33.9	36.4	52.2	[41.2; 63.2]	
Body site of primary						
Axial	147	45.5	46.9	52.8	[43.8; 61.8]	0.322
Extremities	176	54.5	41.5	56.1	[47.8; 64.3]	
Missing data/unknown primary	0/57					
Ulceration of the primary						
Not ulcerated	196	59.3	39.1	61	[52.9; 69.0]	0.013
Ulcerated	135	40.7	48.1	49.1	[39.3; 58.9]	
Missing data/unknown primary	19/57					
Histological subtype of primary						
SSM	121	44.2	39.7	62.2	[52.8; 71.6]	0.013
Nodular	88	32.1	50	50.7	[38.7; 62.6]	
LMM	27	9.8	22.2	73.6	[55.4; 91.8]	
ALM	38	13.9	57.9	33.7	[16.2; 51.1]	
Missing data/unknown primary	49/57					
Clark's level of invasion						
Level I–III	35	13.7	51.4	52.4	[35.3; 69.4]	0.034
Level IV	176	68.8	36.9	64.8	[56.8; 72.8]	
Level V	45	17.6	53.3	34.9	[18.6; 51.2]	
Data Missing/unknown primary	67/57					
Breslow's tumor thickness of primary						
<3 mm	161	52.6	36.6	63.3	[55.1; 71.5]	<0.001
≥3 mm	145	47.4	51.7	45.4	[36.0; 55.8]	
Missing data/unknown primary	17/57					
First occurrence of metastasis						
Initially in patients with known primary	131	34.5	43.5	57.2	[47.6; 68.8]	0.879
During surveillance of stage I/II patients	192	50.5	44.3	52.9	[44.9; 60.9]	
Initially in patients with unknown primary	57	15.0	35.1	54.7	[38.8; 70.5]	
Number of involved nodes						
0	293	77.1	36.5	60.1	[53.6; 66.6]	<0.001
1	39	10.3	61.5	39.9	[22.7; 57.1]	
2–3	31	8.2	64.5	34.9	[16.5; 53.3]	
≥4	15	4.0	73.3	24.4	[0.7; 48.1]	
Missing data	2					

* 95%-CI  = 95% confidence interval; **p-values are results of log rank tests excluding cases with missing values; log rank test excluding cases without lymph node metastasis and those with missing values.

### Survival analysis of patients with in-transit/satellite metastases

**Figure 1:**
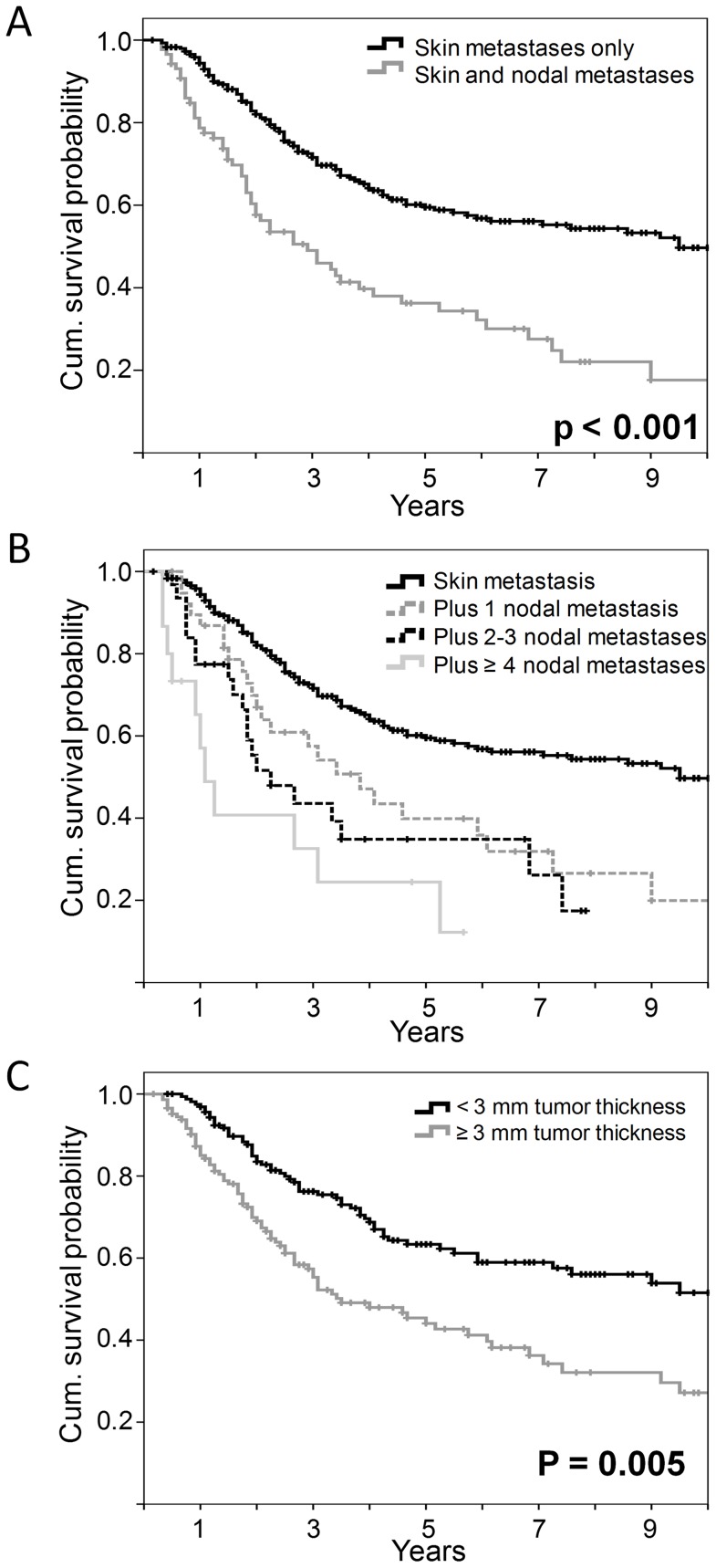
Overall survival of patients with loco-regional skin metastases at the initial diagnosis of metastatic spread. Cumulative survival probabilities are stratified by the presence or absence of additional lymph node metastases (A), the number of additional lymph node metastases (B), or the thickness of the primary melanoma (C) estimated using Kaplan Meier survival analysis. Censored events are indicated by vertical lines.

Five-year survival probability was 60.1% for patients with skin metastases only and 36.3% for those with synchronous nodal metastases (MST 35 months vs. 114 months, log rank p<0.001; Fig. 1A), the number of involved lymph nodes (MST 13, 27 or 46, 114 months for respectively, four or more, two to three, one vs. none involved node, p<0.001: Fig. 1B) and a tumor thickness of 3 mm or more (MST 42 months vs. 123 months, p<0.001; Fig. 1C) were the characteristics with the most impact on survival. Five-year survival rates are presented for all 380 patients with in-transit/satellite metastases in Table 1. Multivariable Cox models identified a strong and consistent correlation with the number of involved lymph nodes (Table 2). The hazard ratios for melanoma-specific death were 2.0 for one, 2.3 for two or three and 3.7 for four or more nodal metastases and 1.7 for patients with primary melanomas with a tumor thickness of at least 3 mm (Table 2). If the number of nodes is neglected in patients with loco-regional skin metastases like in the current AJCC stage III classification algorithm, which only differentiates between presence vs. absence of nodal disease the hazard ratio in case of additional metastastic spread to the lymph nodes is 2.2. Bivariate analysis also showed associations between ulceration, Clark's level, or the histopathological subtype and survival, but these characteristics were no longer found to be statistically significant in the multivariable analysis. No association was found between gender, age or the time of stage III diagnosis in the course of disease and survival in either bi- or multivariable analysis.

**Table 2:** Independent prognostic factors for 377 patients with satellite or in-transit metastases according to the multivariable Cox proportional hazard analysis.

Prognostic factor	Sample size (n = 377*)	% Dead**	Relative risk (95% CI)^#^	p-value
Thickness of primary tumor <3 mm or unknown ≥3 mm	235 (62.3%) 142 (37.7%)	37.0% 52.8%	1 1.7 (1.1, 2.5)	p = 0.008
Positive lymph nodes None 1 involved lymph node 2–3 involved lymph nodes ≥4 involved lymph nodes	292 (77.5%) 39 (10.3%) 31 (8.2%) 15 (4.0%)	36.6% 61.5% 64.5% 73.3%	1 2.0 (1.3, 3.2) 2.3 (1.4, 3.9) 3.7 (2.0, 7.1)	P = 0.002 p = 0.001 p<0.001

#95% CI  = 95% confidence interval; *One patient was censored before any death occurred and was removed from the analysis and two patients were removed because the number of involved lymph nodes was not available; **Disease-specific death; the model was adjusted for the confounding effects of ulceration, whether stage III was diagnosed initially or during follow-up and for missing values for tumor thickness (74 cases; 57 were patients with unknown primary melanoma).

## Discussion

The current study has identified tumor thickness as an independent prognostic factor for melanoma patients with satellite or in-transit metastasis at the time point of first metastatic spread. Furthermore, the already established prognostic impact of the presence or absence of concurrent lymph node involvement was confirmed. To the best of our knowledge, this analysis of 380 patients represents the largest cohort of stage III patients with skin metastases which was analyzed using multivariable survival analysis thus far. The novelty of our study in contrast to others was to include stage I/II patients at the time of the first loco-regional recurrence as well as patients with melanoma of unknown primary tumor whose diagnosis was established by histopathologic examination of skin metastases. Both subgroups generally comprise the majority of stage III melanoma patients with skin metastasis but were only included in limited number of prognostic studies thus far [6], [12]. In our cohort only 34.5% were patients with synchronous primary melanoma and loco-regional skin metastases like analyzed by Balch *et al* [1], while 50.5% were recurrent stage I/II patients and 15% had MUP. The inclusion of recurrent stage I/II patients allowed us to investigate, whether the interval between primary melanoma and appearance of metastases is a prognostic factor, a question that is still controversially discussed [12]–[15]. While we were able to demonstrate an impact on survival according to the interval between primary diagnosis and appearance of distant metastases for stage IV patients [16], we could not observe any difference according to the timing of the first occurrence of metastases as either in stage I/II patients during surveillance, at the time of excision of the primary melanoma or initially in patients with MUP here for stage III patients.

MUP is still an unexplained phenomena. One hypothesis is the regression of the primary melanoma mediated by an effective immune response [17]. Lee *et al* [18] and Prens *et al* [19] both demonstrated a clear survival advantage of patients with MUP after therapeutic lymphadenectomy compared to patients with known primary tumor and explained this observation with a strong endogenous immune response directed against melanoma resulting in both, regression of the primary tumor and a better outcome. Survival was similar of patients with known and unknown primary melanoma in our study and therefore not suggestive for a clinically relevant immune privilege in MUP.

The results of our survival analysis are very similar to that presented in the work of Balch *et al*, who reported 5-year survival rates of 69% for N2c patients and 46% for patients with combined skin and lymph node metastases (N3). The corresponding 10-years survival rates were 52% and 33%, respectively [1]. Survival in our study was slightly worse (5-year survival probability: 60.1%) but almost identical if our analysis was restricted to patients with skin metastasis already existing at the time of initial melanoma diagnosis (5-year survival probability: 69.8%). For patients with combined nodal and skin lesions Balch and co-workers found 5-year survival rates of 46% in contrast to 36% observed here [1]. The discrepancy might reflect differences regarding the average number of involved lymph nodes between both patient cohorts.

In the present study, concurrent lymph node involvement was confirmed as the most important independent prognostic factor for stage III patients with skin metastases. As already considered in the AJCC classification, patients with combined skin and lymph node involvement have a worse prognosis and are classified as stage IIIC compared to those who present exclusively with skin lesions (stage IIIB) [20]. The relative risk to die from melanoma for patients with skin metastasis was 2.2-fold increased in case of concurrent lymph node involvement. We further analyzed if the number of nodes, a characteristic that is considered in the AJCC classification only for patients with metastases confined to the lymph nodes is likewise relevant for those patients with concurrent skin metastasis. Although in our study the number of patients with combined skin and lymph node metastasis was low (n = 87), we could still demonstrate a strong impact of the number of metastatic nodes. The relative risk to die from melanoma was increased according to our multivariable analysis showing a consistent relationship between the risk of melanoma-related death and the number of involved lymph nodes. Consideration of the number of involved lymph nodes instead of the exclusive differentiation into presence vs. absence of nodal disease may therefore allow a more accurate prediction of prognosis for patients with satellite or in-transit metastases.

The second characteristic with independent unfavorable impact on survival was a large tumor thickness of the primary melanoma. This is in accordance with a study of Soong and co-workers who included 236 patients with local skin recurrences and found increasing tumor thickness and an axial localization of the primary melanoma to be predictive of poor survival. Patients with skin metastases already present at the time of initial melanoma diagnosis and those with synchronously occurring lymph node and skin metastases were excluded from this analysis [6]. Messeguer *et al* described 36 stage I/II patients who recurred with loco-regional skin metastases. Survival after recurrence was inversely influenced by a high mitotic activity and a large tumor thickness [7]. Nevertheless, it was an unexpected finding that the thickness of the primary melanoma is superior in predicting prognosis for stage III patients with skin metastasis compared to ulceration, which is associated with survival but does not add an independent predictive impact on prognosis according to our multivariate analysis. The opposite was described for patients with metastases confined to the lymph nodes [2]. Balch and co-workers separately analyzed patients with microscopic lymph node metastases and those with macroscopic disease. In patients with micro-metastasis both ulceration and tumor thickness had a similar impact, while for patients with macro-metastasis tumor thickness lost its independent prognostic role in multivariable analysis [2].

## Conclusion

In addition to lymph node involvement, which was confirmed as the most important prognostic factor, the thickness of the primary tumor was identified to independently contribute to prognosis of melanoma patients with loco-regional skin metastases at the time of the initial metastatic spread. No differences in survival were observed between patients with synchronous skin metastasis already present at the time of the excision of the primary tumor, compared to stage I/II patients with skin recurrence or patients with skin metastasis but unknown primary melanoma. Taking the number of involved lymph nodes into account, as already implemented in the AJCC stage III classification for patients with nodal disease, may allow a more accurate prediction of prognosis for patients with satellite or in-transit metastases.
